# Prediction in the Dynamics and Spoilage of *Shewanella putrefaciens* in Bigeye Tuna (*Thunnus obesus*) by Gas Sensors Stored at Different Refrigeration Temperatures

**DOI:** 10.3390/foods10092132

**Published:** 2021-09-09

**Authors:** Zhengkai Yi, Jing Xie

**Affiliations:** 1 College of Food Science & Technology, Shanghai Ocean University, Shanghai 201306, China; zkyi121121@163.com; 2 Shanghai Professional Technology Service Platform on Cold Chain Equipment Performance and Energy Saving Evaluation, Shanghai 201306, China; 3 National Experimental Teaching Demonstration Center for Food Science and Engineering, Shanghai 201306, China; 4 Shanghai Engineering Research Center of Aquatic Product Processing & Preservation, Shanghai 201306, China

**Keywords:** electronic nose, *Shewanella putrefaciens*, dynamic growth, spoilage prediction, GC-MS

## Abstract

*Shewanella putrefaciens* have a faster growth rate and strong spoilage potential at low temperatures for aquatic products. This study developed a nondestructive method for predicting the kinetic growth and spoilage of *S. putrefaciens* in bigeye tuna during cold storage at 4, 7 and 10 °C by electronic nose. According to the responses of electronic nose sensor P30/2, the fitted primary kinetic models (Gompertz and logistic models) and secondary model (square root function model) were able to better simulate the dynamic growth of *S. putrefaciens*, with high R^2^ and low RMSE values in the range of 0.96–0.99 and 0.021–0.061, respectively. A partial least squares (PLS) regression model based on both electronic nose sensor response values and electrical conductivity (EC) values predicted spoilage of *S. putrefaciens* in bigeye tuna more accurately than the PLS model based on sensor signal values only. In addition, SPME/GC-MS analysis suggested that 1-octen-3-ol, 2-nonanone, 2-heptanone, dimethyl disulfide and methylamine, N, N-dimethyl- are the key VOCs of tuna inoculated with *S. putrefaciens*.

## 1. Introduction

Bigeye tuna (*Thunnus obesus*) is a widely distributed and commercially important fish, favored by consumers because of its good taste and abundant nutrition [1]. However, bigeye tuna is an extremely perishable fish because of microbial spoilage and certain biochemical reactions during processing and storage. Its superior nutritional value and delicious meat make it important to preserve bigeye tuna [2]. Some methods have been used for the preservation of tuna, such as gas packaging, cool store, freezing processing, cryopreservation, etc. Refrigeration is currently an effective storage method used to slow down fish deterioration [3]. The main factor contributing to seafood spoilage during the refrigeration process is the activity of microorganisms. Many studies have reported that the specific spoilage organisms in refrigerated seafood were *Shewanella* spp., *Pseudomonas* spp., *Aeromonas* spp., and *Acinetobacter* spp. [3,4]. *S. putrefaciens* is the main spoilage microorganism of seafood in low-temperature storage, which has the potential for decomposing proteins and trimethyl-amine-N-oxide (TMAO) into ammonia, trimethylamine (TMA), and H_2_S, producing a fishy odor [5]. *Shewanella* and *Pseudomonas* species isolated from spoiled tuna were considered as potential main contributors to spoilage in tuna during refrigerated storage [6]. Many studies have reported that the growth of *S. putrefaciens* may cause tuna to deteriorate during refrigeration [7,8]. Therefore, evaluating the spoilage potential of *S. putrefaciens* is crucial in the spoilage control of tuna at low temperatures.

Spoilage influences shelf life, marketing options, and safety of the product; no one buys spoiled foods nor should spoiled foods be on the market. Researchers usually use many conventional methods to identify fish spoilage, including sensory evaluation techniques, chemical methods [9], and microbiological methods [10]. Although conventional microbiological techniques are economical and simple to perform, these methods are time-consuming and cannot be continuously monitored in real time. The odor is an important indicator to evaluate the freshness of fish. Odors such as amines, ammonia, trimethylamine, and volatile sulfides are produced in marine fish during spoilage [11]. These volatile compounds can be potential indicators of spoilage in marine fish. Gas chromatography/mass spectrometry (GC-MS) has become a standard instrument for quantitative analysis of volatile substances in laboratories [12]. However, it is expensive, time-consuming, and unsuitable for large-scale detection. Moreover, the electronic nose (E-nose), a gas sensor array technology, has become an effective tool for predicting fish spoilage [13]. It can mimic the human olfactory system with sensitive sensors that interact with multiple odors to generate different electrical signals [14]. The E-nose has many advantages in predicting food spoilage, such as portability, non-destructive samples, low cost, short time consumption, and high sensitivity. For example, Semeano et al. [15] developed a system based on a gas sensing gel material coupled with an optical E-nose to detect tilapia deterioration, and the system predicted microbial growth well. In addition to the rapid detection of fish spoilage by the E-nose, fish spoilage can also be predicted using a simple physical index—electrical conductivity (EC). The decomposition of tissues and the outflow of electrolytes during the fish spoilage because of the catabolic activity of microorganisms and the oxidation of the fish body eventually lead to the rise of EC [16]. Heising et al. [17] found that EC values of aqueous solutions of volatile compounds produced by cod were positively correlated with freshness. Other researchers have also found a strong correlation between fish conductivity or electrical impedance and fish spoilage, and EC may be a good predictor of fish spoilage [18,19]. However, few studies have involved the use of electronic noses to predict the dynamic growth of spoilage bacteria in seafood and to predict the spoilage of seafood inoculated with spoilage organisms.

The dynamic growth of *S. putrefaciens* and the spoilage potential in aquatic products are of great importance. It is necessary to find a quick and easy method to study the spoilage of marine fish contaminated by *S. putrefaciens*. At present, there are few studies to predict the spoilage as well as the dynamic growth of specific spoilage bacteria in marine fish at different refrigeration temperatures. For this purpose, sterile tuna blocks were inoculated with *S. putrefaciens* and the changes in the total number of *S. putrefaciens* (TNS), total volatile basic nitrogen, EC, and volatiles at different refrigeration temperatures were investigated. The sensor P30/2 of the E-nose was selected to simulate the primary and secondary dynamic growth of *S. putrefaciens* in tuna. A partial least squares (PLS) regression model based on both E-nose sensor response values and EC values was used to predict the spoilage potential of *S. putrefaciens* in bigeye tuna. Finally, some key volatile organic compounds (VOCs) of tuna inoculated with *S. putrefaciens* were identified by GC/MS, and the correlation between VOCs and gas sensor signal values was analyzed.

## 2. Materials and Methods

### 2.1. Bacterial Strains and Cultural Conditions

*S. putrefaciens* was isolated and identified from spoiled bigeye tuna (Zhejiang Fenghui Ocean Fishing Company Ltd., Zhoushan, Zhejiang, China) and was identified based on 16S rRNA gene sequences, compared in GenBank using the BLAST function. Spoiled bigeye tuna was evaluated by trained panelists from the College of Food Science and Technology, Shanghai Ocean University, based on odor, color, and appearance. Bacteria were stored in tryptone soya broth (TSB) containing 25% glycerin at −80 °C. Before use, *S. putrefaciens* was precultured in brain–heart perfusion infusion (BHI) at 30 °C for 18 h and then cultured in TSB until the maximal concentration (10^8^ CFU/mL).

### 2.2. Sample Preparation and Inoculation

The back muscle blocks of 20 kg tuna were purchased from Zhejiang Fenghui Ocean Fishing Company Ltd., Zhoushan, Zhejiang, China, and divided into rectangular blocks of about 30 g. Three replications were taken for TNS, pH, TVB-N, TMA, and VOC measurements, with ten replications for EC and e-nose measurement. Then, the blocks were sterilized by soaking in 0.5% (*v*/*v*) formalin solution for 40 s and washed in sterile water 2 times. Each sterile block was immersed in a bacterial suspension for 30 s of *S. putrefaciens* inoculation to achieve an inoculum level of 3.0–4.0 log CFU/g. Non-inoculated blocks immersed in sterile normal saline (0.85% NaCl) were used as the control check (CK) group. All samples were packed in a clean tray in a sterile environment and stored at 4, 7, and 10 °C.

### 2.3. Physicochemical Analysis

The physicochemical analysis included the measurement of pH, EC, total volatile basic nitrogen (TVB-N), and trimethylamine (TMA) values.

The EC of tuna blocks was measured using the method described by Yao et al. [19]. Briefly, tuna flesh (10 g) was homogenized and stirred for 30 min in 100 mL of distilled water. The mixture was filtered, and the EC of the filtrate was measured using an EC meter (Mettler Toledo FE20/EL20, Shanghai, China).

The pH value was determined by the method of [20]. The sample treatment was consistent with EC measurement and the pH of the filtrate was measured using a digital pH meter (Cyberscan Model 510; Eutech Instruments Pvt. Ltd., Singapore).

Total volatile basic nitrogen (TVB-N) was performed according to the method of [21]. Five grams of minced tuna flesh was accurately weighed. The TVB-N value was measured by an Automatic Kjeldahl Apparatus (KjeltecTM8400; FOSS Quality Assurance Co., Ltd., Copenhagen, Denmark).

TMA content was determined by Colorimetric Picric Acid Method [22]. Briefly, fish samples and trichloroacetic acid (TCA) were homogenized and mixed. After centrifugation, the supernatant was mixed with formaldehyde, saturated potassium carbonate solution, and toluene. The toluene layer solution and picric acid were mixed thoroughly, and absorbance readings were taken at 410 nm.

Measurements of pH, EC, TVB-N, and TMA were taken every 2 days for 12 days for samples stored at 4 °C, every 2 days for 10 days for samples stored at 7 °C, and every 1 day for 6 days for samples stored at 10 °C.

### 2.4. Microbiological Analysis and Growth Curve Fitting

The total number of *S. putrefaciens* (TNS) was determined by a basic method as described by Qian et al. [23] using iron agar. Briefly, 25 g of tuna flesh were put in 225 mL of sterilized saline water (NaCl, 0.85%, *w*/*v*) and homogenized for 2 min. Then, 0.1 mL of the dilution was spread on iron agar (IA) plates after serial dilution and incubated at 30 °C for 48 h. Black colonies were enumerated in IA plates. Plate counting agar was used to count the total viable count (TVC) in the CK group at 30 °C for 48 h. The measurement cycle of TNS and TVC was the same as the above physicochemical indexes.

#### 2.4.1. Primary Models

The primary models, namely, Gompertz and logistic models, were used to simulate the growth curves of *S. putrefaciens* in tuna. They are represented by the following equations according to Gibson et al. [24]:

(1) $$N=N_{0}+\left(N_{max}-N_{0}\right)\times \exp\left(-\exp\left(\mu_{max}\times \frac{e}{N_{max}-N_{0}}\times \left(\lambda -t\right)+1\right)\right),$$

(2) $$N=N_{0}+\left(N_{max}-N_{0}\right)/\left(1+\exp(\mu_{max}\times (\lambda -t\right))$$

where *N* is the cell concentration (log CFU/g) at time t, *N*_0_ and *N_max_* represent the initial and maximum cell numbers (log CFU/g) of *S. putrefaciens*, respectively. *λ* is the lag time (h), *t* is real time, and *μ_max_* represents the maximum growth rate (per h).

#### 2.4.2. Secondary Models

To describe the temperature effect on *μ_max_* and *λ*, a second model (square root model) was used as follows:

(3) $$\sqrt{1/\lambda }=a_{1}\times \left(T-T_{min1}\right),$$

(4) $$\sqrt{\mu_{max}}=a_{2}\times \left(T-T_{min2}\right)$$

where *a*_1_ and *a*_2_ are regression coefficients; *T* is the real temperature in °C; *T_min_*_1_ and *T_min_*_2_ are the theoretical minimum growth limits in °C.

### 2.5. E-Nose Analysis

Detection of the volatile compounds of the tuna flesh was performed by an electronic nose (E-nose, Fox 4000 Alpha-MOS, France). The 18 sensors are designed as follows: LY2/LG “Chlorine, fluoride, nitrogen oxide”, LY2/G “Ammonia, amines, carbon oxides”, LY2/AA “Alcohol”, LY2/gCTL “Hydrogen sulphide”, LY2/gCT “Propane and butane”, T30/1 “Polar compounds, hydrogen chloride”, P10/1 “Hydrocarbon, ammonia, chlorine”, P10/2 “Methane and ethane”, P40/1 “Fluoride and chlorine”, T70/2 “Toluene and xylene”, PA/2 “Ethanol, ammonia, amines”, P30/1 “Hydrocarbon”, P40/2 “Chlorine and fluoride”, P30/2 “Hydrogen sulfide and ketones” T40/2 “chlorine”, T40/1 “Fluoride”, TA/2 “Alcohol”. A total of 2.0 g of flesh was placed in a 10 mL vial for 10 min at 50 °C to generate balanced headspace samples. The gas flow rate was 2.5 mL/min, and the sensor cleaning time was 8 min. Then, the sensor response of the E-nose was determined as G/G0 (G0 and G represent the conductivity of the sensor exposed to the zero gas and sample gas). The measurement cycle of the E-nose of the sample was the same as above.

### 2.6. Headspace Solid Phase Microextraction Gas Chromatography/Mass Spectrometry (SPME-GC/MS) Analysis

According to the method of Li et al. [25] with minor modifications, 2 g of the minced sample was placed into a 20 mL glass vial and equilibrated at 40 °C for 20 min. The SPME extraction fiber was exposed to headspace for 30 min. Gas chromatography coupled with mass spectrometry (GC-MS) was used to measure the volatiles in bigeye tuna. The carrier gas was helium (high purity 99.999%), with a constant flow rate of 1 mL/min. The oven temperature program was as follows: initial temperature of 40 °C for 5 min, 5 °C/min to 120 °C, then 10 °C/min to 250 °C, and held for 5 min. Next, the volatiles were transferred to the MS system, MS source and quadrupole: 230 and 150 °C, respectively. Mass spectra were obtained within the mass range of 20–400 m/z at 70 eV. The VOCs of samples stored at 4 and 10 °C were measured. The samples at 4 °C were measured on days 4, 8 and 12, and the samples at 10 °C were measured on days 2, 4 and 6.

### 2.7. Statistical Analysis

The measurement experiments of pH, TVB-N, TMA, TNS, and SPME-GC/MS were repeated three times, and the EC and E-nose experiments were repeated ten times. The data of VOCs were expressed as the mean ± standard deviation. The growth kinetic model of *S. putrefaciens* was fitted using MATLAB 2017b (Math Works Inc., Natick, MA, USA). Pearson correlation analysis was used to evaluate the correlation between sensor response values and TNS to select appropriate sensors for predicting the dynamic growth of *S. putrefaciens.* Duncan’s test and Pearson correlation coefficient were performed using SPSS 19.0 (SPSS Inc., Chicago, IL, USA). PLS regression was used to predict the spoilage potential of *S. putrefaciens* in tuna including TVB-N, TMA, and TNS. It is well known that the predictive performance of the calibration model cannot be determined merely by the internal validation but should also be externally validated based on predictions for samples not included in the calibration test. Data measured by the E-nose and electric conductivity meter were randomly divided into a training set (developing fitted models) and a testing set (validating models) in the ratio of 7:3. The training set of response values of the E-nose sensors was used as the data for building models, and the testing set was used as independent data to verify the accuracy of models. The data of pH, TVB-N, TMA, TNS, and SPME-GC/MS were not divided into training and validation sets. This was mainly because the data obtained from the E-nose and EC meter were used as source data of rapid detection to predict spoilage indicators (including TVB-N, TMA, TNS, and SPME-GC/MS) in the samples. Therefore, the spoilage indicators were used as a predicted object without being divided into training and validation sets (each indicator was considered as a whole). In addition, TNS was also one of the predicted indicators and was not divided into training and validation sets for the growth curve fitting. The model accuracy was evaluated by determination coefficient (R^2^) and root-mean-square error (RMSE). RMSE was calculated as follows:

(5) $$RMSE=\sqrt{\frac{1}{n}\overset{n}{\underset{i=1}{\sum }}{\left(y_{m}-y_{p}\right)}^{2}}$$

where *y_m_* and *y_p_* are measured and predicted values.

## 3. Results and Discussion

### 3.1. TVB-N and TMA

**Figure 1:**
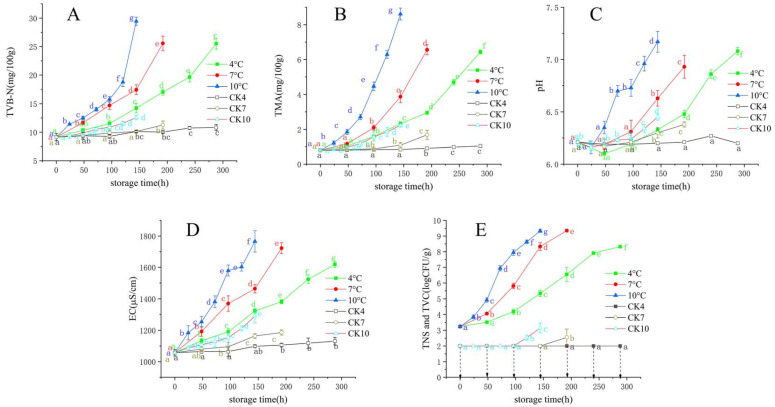
Changes in total volatile base nitrogen (TVB-N) (**A**), trimethylamine (TMA) (**B**), pH (**C**), Electrical conductivity (EC) (**D**), and total number of *S. putrefaciens* (TNS) of inoculation groups and total viable count (TVC) of control check (CK) groups (**E**) in bigeye tuna stored at different temperatures (each point is the mean value of three determinations). CK4, CK7, and CK10 are CK groups stored at 4, 7, and 10 °C. a–g in the same column with different superscripts are significantly different (*p*  <  0.05). The arrow indicates that the viable count was below 2.0 log CFU/g.

Changes in the TVB-N content of bigeye tuna blocks are shown in Figure 1A. The TVB-N values increased in inoculated tuna blocks throughout the storage period at different temperatures. TVB-N values of all groups showed a slow increase followed by a rapid increase. This result was consistent with Li et al. [26], who reported that when *S. putrefaciens* was inoculated into blunt snout bream flesh stored at 4 °C for 14 days, TVB-N values of samples were slow on the first 8 days, but increased rapidly on the last 4 days.

The TMA value increased gradually during storage at 4, 7, and 10 °C (Figure 1B). Similar to the variation pattern of TVB-N values, the TMA values of inoculated tuna samples stored at 10 °C for 6 days were higher than those stored at 4 and 7 °C. This result indicated that temperature is a vital factor for microbial activity. The possible reason for the significant difference between the results for the CK and inoculated group may be that the production of TMA was promoted by *S. putrefaciens* in the inoculated samples, and the type of bacteria determined the ability to produce TMA. The TMA of samples increased exponentially with storage time, which was in agreement with [27], who reported that the change in TMA of yellowfin tuna fitted an exponential growth during the refrigeration.

### 3.2. pH and EC

The changes in the pH of aquatic products were closely associated with a series of chemical reactions caused by endogenous enzymes and microorganisms [28]. Changes in pH values in tuna samples at different temperatures are presented in Figure 1C. The initial pH value was 6.21 with a decreasing and then an increasing trend. A decrease in pH of the samples was due to the generation of lactic acid and the release of inorganic phosphate by decomposition of ATP [1], while the increase in the pH value was related to the release of alkaline decomposition products, such as histamine and TMA produced by spoilage microorganisms [29]. The pH of the sterile fish blocks (CK) stored at 4 °C fluctuated around 6.21, while those stored at 7 and 10 °C increased slowly. This may be due to the growth and metabolism of residual microorganisms in the CK group, and a similar phenomenon was observed in TVB-N and TMA.

EC can be used to detect meat quality from the efflux and excessive breakdown of body fluids from fish tissue during storage. Variations in EC value during cold storage are presented in Figure 1D. Initially, the EC in samples was 1057 μS/cm, and the EC of each group of samples increased significantly during storage. The change rate of EC values was higher for samples stored at higher temperatures. The significant increase in EC may be due to the autolysis of tuna cells after death and the decomposition of various nutrients in the cells into ions and small molecule metabolites with electrical conductivity under the action of enzymes and microorganisms, which enhances the electrical conductivity of the cell leachate [30]. Similar to the TVB-N and TMA curves, EC values increased slowly in the CK groups stored at 4, 7, and 10 °C.

### 3.3. Results of the E-Nose Analysis

**Figure 2:**
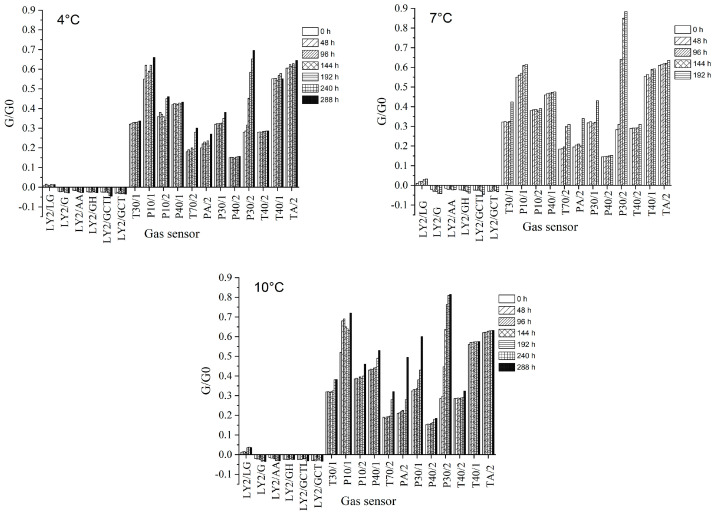
Average responses of 18 sensors in tuna samples inoculated with *S. Putrefaciens* at different temperatures during storage.

As shown in Figure 2, odor maps were obtained with the E-nose from samples stored at 4, 7 and 10 °C. Response values from each sensor represent the average of 10 measurements. The E-nose responses of bigeye tuna samples stored at different storage temperatures showed a similar trend. Furthermore, sensors T70/2, PA/2, P30/1, and P30/2, which were sensitive to aromatic compounds, amines, hydrocarbons, and hydrogen sulfide, increased significantly at all storage temperatures. However, the signal values of sensors PA/2 and P30/2 increased more remarkably at 7 and 10 °C than those at 4 °C, indicating that storage temperature was an essential factor affecting the production of some compounds of tuna in storage. This finding was consistent with other studies that found temperature to be an important factor influencing the production of metamorphic substances in fish during refrigeration [31]. Changes in sensor signal values over time at different temperatures may be due to an increase in volatile gas concentrations and the production of new gas species [32], which may be related to the growth temperature and the number of *S. putrefaciens*.

**Figure 3:**
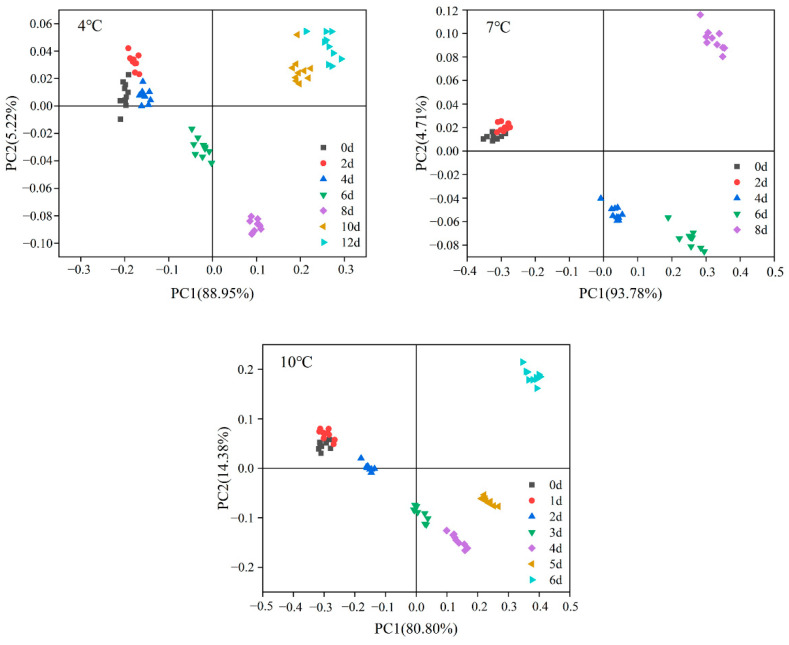
PCA score plots based on electronic nose measurements of tuna inoculated with *S. Putrefaciens* with different incubation times.

The corresponding values from the sensor arrays were explored to determine the differences in volatilization patterns of tuna during different cold storage periods using principal component analysis (PCA). To test whether the electronic nose could distinguish samples with different storage times, PCA was employed to investigate the feasibility of distinguishing tuna inoculated with *S. putrefaciens* sampled at different times and temperatures based on E-nose signals. As shown in Figure 3, the differences in tuna with different storage times can be represented using two main principal components (PCs), which accounted for 88.95% (4 °C), 93.78% (7 °C), and 80.8% (10 °C) of the total variance in PC1 and 5.22%, 4.71%, and 14.38% in PC2. Regarding the samples stored at 4 °C, the data points of groups 0, 2 and 4 d were placed in the first cluster, whereas the other groups were separated into another three clusters (6, 8 and 10–12 d). The data points of groups at 0, 2, and 4 d were similar, indicating that the odor profiles of the early contaminated samples were relatively similar. But the samples at 6, 8, 10, 12 d were clearly classified by PCA. For the samples stored at 7 °C, groups at 0 and 2 d were mixed in a cluster, and groups at 4 and 6 d had a clear right downshift along the ordinate (PC 1), located into the second cluster. Groups at 8 h were separated into another cluster along the abscissa (PC2), located away from other clusters. In 10 °C groups, the data of groups at 0–2 d located into the first cluster, and another two clusters contained 3–5 d and 6 d, respectively. For samples stored at 7 and 10 °C, PCA analysis indicated that the data points of the samples at the beginning of storage (0 and 2 d for 7 °C, 0 and 1 d for 10 °C) overlapped and were initially difficult to distinguish but could be distinguished at later time points. The samples stored at 4, 7, and 10 °C showed that the E-nose based on PCA analysis poorly identified very early contaminated samples but had good overall resolution for tuna inoculated with *S. putrefaciens*. Therefore, E-nose could be employed as a promising approach to realize the prediction of dynamics and spoilage of *Shewanella putrefaciens.*

### 3.4. Dynamic Growth of S. putrefaciens in Tuna

The changes in the viable count of bigeye tuna are shown in Figure 1E. Gompertz and logistic models were fitted to the dynamic growth of *S. putrefaciens*. As shown in Table 1, the high R^2^ and low RMSE values indicated a good fit of models, with ranges of 0.985–0.999 and 0.0654–0.283, respectively. The lag time of the lag phase (λ) and the maximum specific growth rate (μ_max_) were two vital parameters for predicting the growth of microorganisms [33]. λ is especially vital to monitor food microorganisms and ensure food safety [34]. In our work, it was clear that the value of λ for *S. putrefaciens* decreased with increasing temperature, while μ_maxe_ was the opposite (Table 1). We observed that the storage temperature had a significant effect on the growth of *S. putrefaciens*, with slow growth rates at 4 and 7 °C, while a significant growth was observed at 10 °C based on λ_e_ and μ_maxe_ values. The growth of *S. putrefaciens* in tuna was in agreement with previous studies [35]. In addition, the initial colony count (N_0_) of *S. putrefaciens* in tuna ranged from 2.3 to 3.3 log CFU/g; the value of the maximum colony count (N_max_) peaked between 8.5 and 10.4 log CFU/g, according to two growth models.

**Figure 4:**
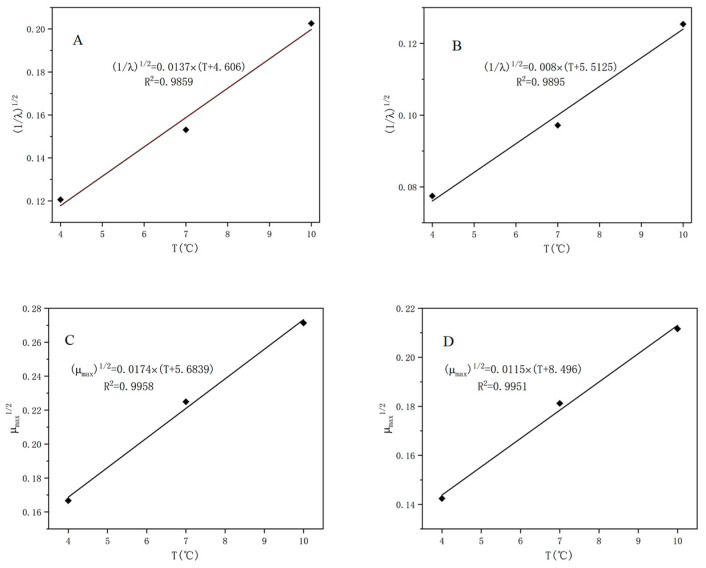
The secondary growth models of *S. putrefaciens* based on modified Gompertz (**A**,**C**) and logistic model (**B**,**D**).

However, the primary models cannot estimate the effect of temperature on the growth of *S. putrefaciens* in tuna, but the secondary model can evaluate it. Therefore, the square root model was used to describe the relationship between the growth parameters ($\sqrt{\mu_{maxe}}$ and $\sqrt{\lambda e^{-1}}$) and the storage temperature for the microbe. As shown in Figure 4, the results showed a strong linear correlation between the kinetic parameters and the storage temperature, with R^2^ values higher than 0.98. Our study also predicted the minimum growth temperature (T_min_) of *S. putrefaciens* in tuna in the range of −8.5 to −4.6 °C based on a secondary model, which was slightly higher than −11.4 °C predicted by [36]. This may be due to the difference in fish samples and handling.

### 3.5. Modeling the Kinetics of S. putrefaciens in Tuna with E-Nose Sensors

In this study, Pearson correlation analysis was used to determine the correlation between the signal values of sensors and the number of *S. putrefaciens* colonies in tuna. Finally, sensor P30/2 was selected (data not shown). In addition, the relatively high response values of sensor P30/2 and the considerable variation with storage time indicated its sensitivity to tuna samples during storage. Therefore, in the same way, an attempt was made to simulate the growth of *S. putrefaciens* by fitting the response of the sensor with Gompertz and logistic functions. Sensor P30/2 responses were fitted via Gompertz and logistic models to simulate *S. putrefaciens* growth according to the training set, and the validation set was used to verify the quality of the prediction models. The parameters of the generated mathematical equations are shown in Table 2, and the λ_e_ and μ_maxe_ of CFU were derived from Table 1. The sensor fitted both models well, with high R_c_^2^ and low RMSE_c_ values, in a range of 0.971–0.994 and 0.0208–0.0472, respectively. Validated with the testing set, the fitting models were credible, with similar high R_p_^2^ and low RMSE_p_ values of 0.963–0.987 and 0.0301–0.0613, respectively.

In addition, the P/30 λ_s_ and μ_maxs_ values obtained by the sensor based on the logistic model fit were closer to the results obtained from the actual growth model compared to the Gompertz model (Table 2). It indicated that the logistic model was more suitable for predicting of the growth of *S. putrefaciens* by the gas sensor in our study, which was different from the result reported by Gu et al. [34]. This difference may be due to differences in the gas sensors selected and strains. Kinetic parameters (λ_s_ and μ_maxs_) are of special interest in predicting microbiology and are of high practical value in monitoring food quality and safety [37]. A high correlation coefficient (r) was obtained by comparing the growth fit models generated by sensor P/30 with the models in Table 1, which indicated that the response changes of sensor P/30 to the sample were similar to the growth of *S. putrefaction*. Microbial kinetic models according to microbial counting methods often have difficulty in obtaining λ_s_ and μ_maxs_ of microbial growth because of long training times [38]. Our study showed that a microbial odor response sensor may be used to simulate the dynamics of *S. putrefaciens* in tuna, but this requires more experimental verification.

**Figure 5:**
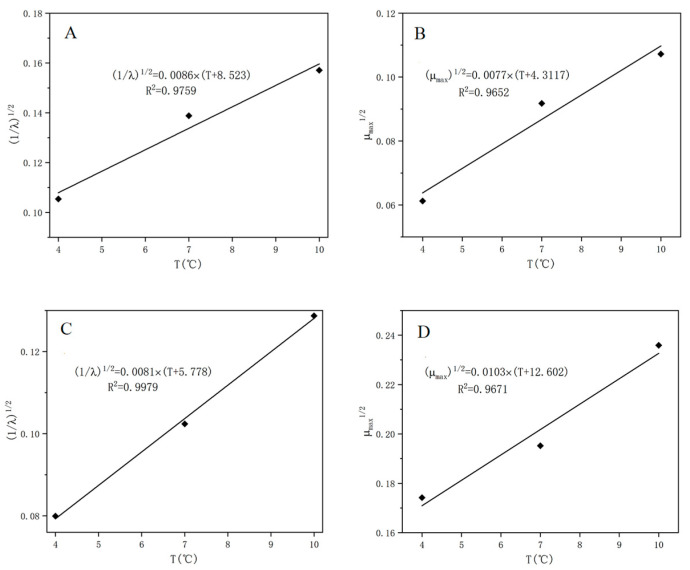
The secondary growth model of *S. putrefaciens* based on the response values of selected sensors P30/2. (**A**,**B**): Square root model based on Gompertz model; (**C**,**D**): Square root model based on logistic model.

A secondary model of the gas sensor P30/2 was also established and showed a good fit with R^2^ in the range of 0.965–0.998, and the linear relationship based on logistic equation was slightly stronger than that of the Gompertz equation for λ_s_ and μ_maxs_ (Figure 5). Most studies predicted the number of microorganisms based on the chemometric method using multiple E-nose sensors [31,39,40], but rarely predicted the dynamic growth of spoilage microorganisms in aquatic products by a single sensor. In our study, primary and secondary kinetic models of *S. putrefaciens* in tuna were fitted by a single sensor, and the kinetic parameters were obtained.

### 3.6. Prediction of the Spoilage of S. putrefaciens in Tuna

To predict the spoilage level of inoculated bigeye tuna, the PLS algorithm was used to evaluate the correlation between the E-nose responses and spoilage indicators (TVB-N, TMA, and TNS) of the tuna. In addition, the changes in tissues caused by fish spoilage can be reflected by electrical conductivity [41,42], and the measurement method of EC is relatively simple. Therefore, the sensor responding values of E-nose and EC values were also combined to predict the spoilage of tuna. For PLS regression modeling, E-nose and EC measurement values from each group were randomly separated into two sets: seven samples used as the calibration set for and the remaining three samples as the prediction set. The leave-one-out cross-validation method was used to improve the accuracy of the PLS model.

**Figure 6:**
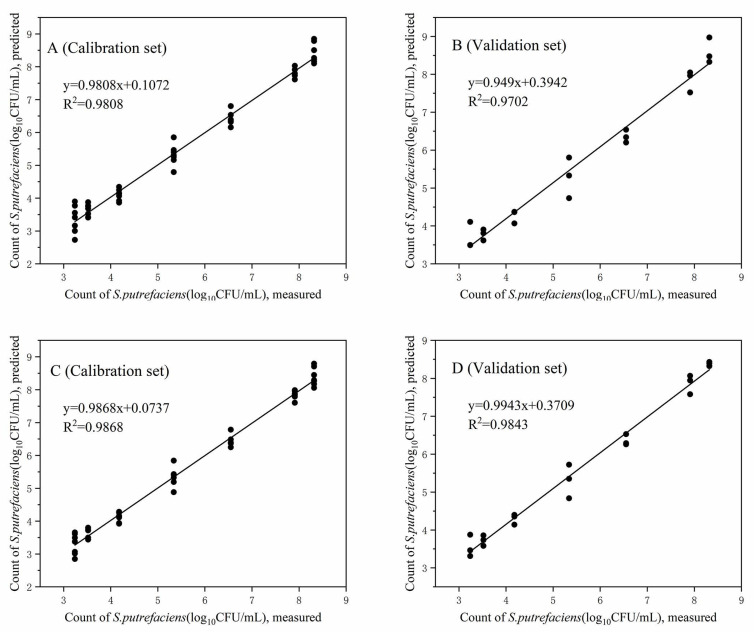
Reference measured data versus predicted data from the PLS models for prediction of the total number of *S. putrefaciens* (TNS) in tuna stored at 4 °C. (**A**,**B**): PLS models for calibration and validation set without EC values; (**C**,**D**): PLS models for calibration and validation set with EC values.

Scatter graphs of the count of *S. putrefaciens* in tuna stored at 4 °C based on the PLS with and without EC values are given in Figure 6. The R^2^ and RMSE values between the predicted and experimental values are shown in Table 3. It is well known that the key to evaluating the quality of a predictive model is not only internal validation (calibration) but also external validation of samples not included in the calibration set [43]. In the two sets, the predictions of the PLS models with and without EC values performed well, and the PLS model with EC values was better than those without EC values, except for the prediction of TMA values for tuna stored at 10 °C (but the difference was not significant). The reason for this result was that the accuracy of the PLS prediction model improved by adding the index (EC) that had a great correlation with the prediction object [44].

### 3.7. Volatile Compounds in Tuna Samples According to HS-SPME/GC-MS

In this work, a total of 30 VOCs were detected in the control and inoculated groups (Table 4). The changing trend of VOCs at 10 °C was greater than that at 4 °C, and various VOCs in this study were previously reported to be products of protein or lipid oxidation metabolism [45]. To exclude the effect of oxidation of the fish itself on VOCs, this study also measured the VOCs produced by non-inoculated tuna blocks during storage.

The increasing tendency of 1-Penten-3-ol, 1-Octen-3-ol, and 2-Hexen-1-ol, (Z)- was observed in tuna during storage, and their increase was associated with auto-oxidative derivatization of polyunsaturated fatty acids [46]. Ethanol was present only in the early stages of tuna storage (day 0, day 4–4, and day 2–10) and was not detected as the storage period increased, which was similar to the results of Liu et al. [10]. This may be related to the fact that the metabolic process of *Shewanella* does not produce ethanol but can use it [4]. In addition, hexanal, heptanal, octanal, nonanal, and propanal were detected at the early stage of storage, and the above substances were confirmed to be produced by fat oxidation and had a fishy odor. The content of hexanal increased with storage time and storage temperature, which may indicate that the fat in tuna was oxidized. The changes of aldehydes reflect the degree of oxidation of polyunsaturated fatty acids such as linoleic acid in bigeye tuna, which can be used as a basis for judging the freshness of tuna. Ketones including 2-nonanone, 2-undecanone, and 2-heptanone were significantly higher in inoculated tuna compared to the control group. These ketones may originate from lipolysis and dehydrogenation by spoilage bacteria [47], which explains the low ketone content of fresh fish samples (day 0). Some hydrocarbons were also detected in this study, which were mainly derived from the decomposition of alkoxy radicals of fatty acids. Various hydrocarbons were present in the volatiles of crustaceans and fish, but they had a high threshold and made little contribution to the flavor of bigeye tuna [48]. Methylamine, N, N-dimethyl-(so-called trimethylamine) were detected only after 8–12 days at 4 °C and 4–6 days at 10 °C, which may be because of higher concentrations of TMA in the inoculated tuna during the late storage period. Dimethyl disulfide was detected in the inoculated tuna at the late storage period. This compound derived from the methionine catabolism that was produced by *S. putrefaciens* [49].

### 3.8. Relationship between E-Nose Results and VOCs

**Figure 7:**
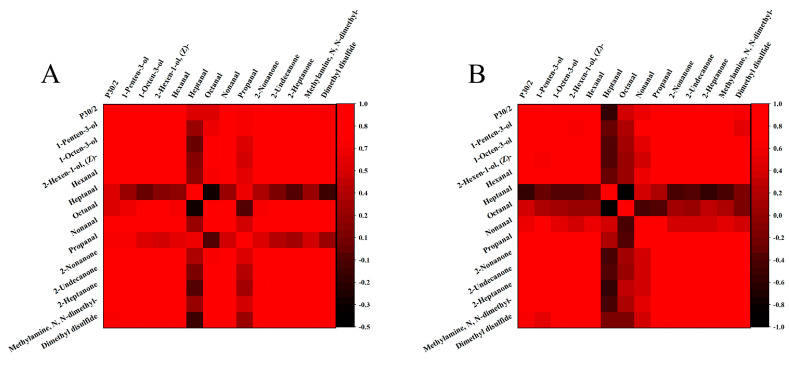
Correlations between sensor responses and GC-MS data (**A**): 4 °C; (**B**): 10 °C.

In this work, characteristic VOCs, including alcohols, aldehydes, ketones, amines, and sulfide compounds, played a significant role in distinguishing tuna samples infected with *S. putrefaciens* by GC-MS at different temperatures. Sensor P30/2 was sensitive to hydrogen sulfide and ketone. Comparing the VOCs with the sensor P30/2, the presence of alcohols, ketones, amines, and sulfide compounds had a significant impact on the sensor response. The significantly increased response values of the P30/2 were consistently correlated with the increased concentrations of ketones and sulfides. In addition, the relationship between sensor responses and VOCs was investigated by Pearson correlation coefficients (Figure 7). Correlation coefficient results indicated that the sensor responses were positively correlated with 1-octen-3-ol, 2-nonanone, 2-heptanone, dimethyl disulfide, and methylamine, N, N-dimethyl-, which revealed that the P30/2 was sensitive to representative VOCs of tuna inoculated with *S. putrefaciens*. In particular, dimethyl disulfide, and methylamine, N, N-dimethyl-, as the characteristic volatile compounds of *S. putrefaciens* [10], contained high content, which contributed significantly to the high signals of P30/2 to inoculated tuna. Therefore, the selected sensor could be used to discriminate tuna infected with *S. putrefaciens* through the specific response to characteristic VOCs.

## 4. Conclusions

This work demonstrated that the growth of *S. putrefaciens* in tuna samples stored at 4, 7, and 10 °C was consistent with two primary kinetic models (Gompertz and logistic), with high R^2^ and low RMSE values in the range of 0.985–0.999 and 0.0654–0.283, respectively, as well as a secondary kinetic model with high R^2^ values in the range of 0.9859–0.9958. The selected sensor P30/2 accurately predicted the dynamic growth of *S. putrefaction*. In addition, the secondary model was used to characterize the relationship between the storage temperature of the samples and the growth kinetic parameters of *S. putrefaciens*. The secondary model fitted with the sensor P30/2 accurately estimated the influence of temperature on the kinetic parameters of *S. putrefaciens* and the minimum growth temperature range of *S. putrefaciens*. The PLS model based on the E-nose response values with the EC values was more accurate than the model without the EC values in predicting the spoilage of tuna inoculated with *S. putrefaciens.* Based on the GC-MS analysis, several alcohols, ketones, amines, and sulfide compounds, especially 1-octen-3-ol, 2-nonanone, 2-heptanone, dimethyl disulfide, and methylamine, N, N-dimethyl- were determined as characteristic VOCs in tuna infected with *S. putrefaciens* stored at 4 and 10 °C. These results revealed that the E-nose can have a wide range of applications for predicting the growth of spoilage microorganisms and performing a quantitative analysis of spoilage in tuna.

## Figures and Tables

**Table 1:** The primary growth models of *S. putrefaciens* in tuna at different temperatures based on modified Gompertz and logistic equation by CFU.

Fitted Models	T/°C	Equations	λ_e_ (h)	μ_maxe_ (h^−1^)	No	N_max_	R^2^	RMSE
Gompertz	4	f(x) = 2.276 + 6.222 × exp(−exp(0.07551/6.222 × (68.8 − x) + 1))	68.8	0.02778	2.276	8.498	0.997	0.101
	7	f(x) = 3.292 + 7.149 × exp(−exp(0.1375/7.149 × (42.67 − x) + 1))	42.67	0.05059	3.292	10.441	0.994	0.1387
	10	f(x) = 3.252 + 6.331 × exp(−exp(0.2/6.331 × (24.36 − x) + 1))	24.36	0.07358	3.252	9.583	0.986	0.283
Logistic	4	f(x) = 3.053+5.783/(1 + exp(0.02028 × (166.6 − x)))	166.6	0.02028	3.053	8.836	0.998	0.0763
	7	f(x) = 3.092 + 6.657/(1 + exp(0.03285 × (105.9 − x)))	105.9	0.03285	3.092	9.749	0.999	0.0654
	10	f(x) = 2.83 + 6.48/(1 + exp(0.04478 × (63.6 − x)))	63.6	0.04478	2.83	9.31	0.985	0.167

**Table 2:** Parameters of dynamic growth models of *S. putrefaciens* in tuna stored at different temperatures based on P30/2 by modified Gompertz and logistic equations.

Model	T/°C	Equations	Training Set		Testing Set		Sensor		CFU		r
			Rc^2^	RMSEc	Rp^2^	RMSEpc	λ_s_ (h)	μ_maxs_ (h^−1^)	λ_e_ (h)	μ_maxe_ (h^−1^)	
Gompertz	4	f(x) = 0.280 + 0.401 × exp(−exp(0.0102/0.4023 × (90.05 − x) + 1))	0.978	0.0261	0.968	0.0307	90.05	0.003753	68.8	0.02778	0.986
	7	f(x) = 0.282 + 0.6125 × exp(−exp(0.0229/0.6125 × (52.27 − x) + 1))	0.994	0.0208	0.985	0.0334	52.27	0.008425	42.67	0.05059	0.976
	10	f(x) = 0.2872 + 0.509 × exp(−exp(0.03126/0.509 × (40.51 − x) + 1))	0.983	0.0341	0.987	0.0324	40.51	0.01150	24.36	0.07358	0.986
Logistic	4	f(x) = 0.2598 + 0.4307/(1 + exp(0.03033 × (156.50 − x)))	0.971	0.0315	0.965	0.0329	156.50	0.03033	166.6	0.02028	0.996
	7	f(x) = 0.2352 + 0.658/(1 + exp(0.03812 × (95.24 − x)))	0.977	0.0472	0.963	0.0613	95.24	0.03812	105.9	0.03285	0.995
	10	f(x) = 0.2629 + 0.5553/(1 + exp(0.04864 × (60.36 − x)))	0.978	0.0391	0.987	0.0301	60.36	0.04864	63.6	0.04478	0.999

**Table 3:** Calibration and validation results in tuna stored at different temperatures based on the PLS model with and without EC values.

Indicators	Temperatures (°C)	PLS without EC Values				PLS with EC Values			
		Calibration Set		Validation Set		Calibration Set		Validation Set	
		R_c_^2^	RMSE_c_	R_v_^2^	RMSE_v_	R_c_^2^	RMSE_c_	R_v_^2^	RMSE_v_
TVB-N (mg/100 g)	4	0.9713	0.9327	0.9775	0.8673	0.9812	0.7519	0.9902	0.6448
TMA (mg/100 g)		0.99	0.1932	0.9862	0.2425	0.9905	0.189	0.988	0.2237
TNS (log_10_ CFU/mL)		0.9808	0.2679	0.9702	0.3589	0.9868	0.222	0.9843	0.2653
TVB-N (mg/100 g)	7	0.9874	0.6461	0.9863	0.6981	0.9925	0.4966	0.9919	0.5053
TMA (mg/100 g)		0.9956	0.1418	0.9851	0.322	0.9962	0.1326	0.9966	0.1292
TNS (log_10_ CFU/mL)		0.9956	0.1594	0.9923	0.225	0.9957	0.1565	0.995	0.1742
TVB-N (mg/100 g)	10	0.9932	0.5411	0.9896	0.6755	0.9958	0.4485	0.9963	0.4214
TMA (mg/100 g)		0.9897	0.276	0.9871	0.3478	0.9876	0.3020	0.9826	0.3837
TNS (log_10_ CFU/mL)		0.9857	0.2735	0.969	0.4185	0.9963	0.1396	0.9864	0.2705

**Table 4:** Volatile organic compounds (VOCs) in tuna at 4 and 10 °C. Day 4–4, day 8–4, and day 12–4: inoculated tuna stored for 4, 8, and 12 days at 4 °C, respectively. Day 2–10, day 4–10, and day 6–10: inoculated tuna stored for 2, 4, and 6 days at 10 °C, respectively. CK 12–4 and CK 6–10: non-inoculated tuna stored for 12 and 6 days at 4 and 10 °C, respectively.

VOCs	Relative Concentration (Area 10^−6^)								
	Day 0	Day 4–4	Day 8–4	Day 12–4	CK 12–4	Day 2–10	Day 4–10	Day 6–10	CK 6–10
Alcohols									
1-Penten-3-ol	3.01 ± 1.13	3.56 ± 0.56	3.89 ± 1.36	4.65 ± 1.98	4.54 ± 0.64	6.89 ± 0.98	8.99 ± 2.36	9.29 ± 1.99	7.98 ± 2.36
1-Octen-3-ol	7.28 ± 1.86	21.69 ± 0.98	20.79 ± 3.25	46.13 ± 6.56	20.36 ± 2.56	18.61 ± 0.56	35.79 ± 2.11	66.98 ± 5.24	35.98 ± 4.65
Ethanol	9.65 ± 1.23	1.23 ± 0.21	ND	ND	ND	2.23 ± 0.56	ND	ND	ND
1-Hexanol	12.56 ± 0.33	6.22 ± 0.65	2.12 ± 0.32	ND	1.36 ± 0.05	3.56 ± 0.65	ND	ND	2.98 ± 0.06
(3-Methyl-oxiran-2-yl)-methanol	ND	0.66 ± 0.23	0.68 ± 0.24	1.65 ± 0.66	0.12 ± 0.04	ND	2.38 ± 0.56	3.01 ± 0.21	0.22 ± 0.08
2-Hexen-1-ol, (Z)-	ND	0.54 ± 0.04	1.89 ± 0.03	5.65 ± 0.69	ND	ND	2.32 ± 0.05	9.28 ± 0.32	ND
2-Nonen-1-ol	0.4 ± 0.02	1.25 ± 0.11	0.65 ± 0.01	0.33 ± 0.02	0.43 ± 0.03	0.54 ± 0.06	1.56 ± 0.02	0.51 ± 0.03	0.36 ± 0.02
Aldehydes									
Hexanal	7.86 ± 0.65	19.23 ± 1.89	33.21 ± 3.12	64.93 ± 5.36	13.54 ± 2.36	22.52 ± 1.35	35.26 ± 3.22	71.26 ± 5.62	21.22 ± 2.56
Heptanal	4.22 ± 0.12	4.12 ± 0.63	9.52 ± 1.32	5.24 ± 0.97	3.22 ± 0.86	8.79 ± 1.22	1.23 ± 0.06	3.54 ± 0.07	1.98 ± 0.21
Nonanal	2.21 ± 0.08	2.28 ± 0.21	4.60 ± 0.11	8.21 ± 0.99	3.21 ± 0.07	18.47 ± 2.36	12.11 ± 2.65	15.89 ± 3.06	11.21 ± 2.10
Propanal	2.35 ± 0.09	5.61 ± 0.46	7.22 ± 1.23	6.11 ± 0.12	2.12 ± 0.33	5.31 ± 0.22	4.33 ± 1.11	6.85 ± 0.64	5.11 ± 0.21
2-Decenal, (E)	ND	3.12 ± 0.18	2.47 ± 0.21	1.56 ± 0.06	ND	ND	0.43 ± 0.05	2.55 ± 0.65	0.35 ± 0.05
2-Nonanal, (E)-	ND	0.22 ± 0.03	3.56 ± 0.24	4.54 ± 0.68	3.72 ± 0.21	0.31 ± 0.05	5.33 ± 0.98	5.99 ± 1.33	3.56 ± 0.09
2-Dodecanal, (E)-	ND	ND	1.263 ± 0.05	2.112 ± 0.32	4.6 ± 0.23	5.68 ± 1.22	4.22 ± 0.09	3.68 ± 1.21	2.23 ± 0.21
Decanal	0.65 ± 0.02	1.36 ± 0.05	ND	ND	2.55 ± 0.65	1.55 ± 0.96	2.36 ± 0.12	1.32 ± 0.08	1.86 ± 0.09
Octanal	3.86 ± 0.33	1.69 ± 0.35	0.98 ± 0.04	9.98 ± 2.65	2.65 ± 0.22	1.98 ± 0.21	5.65 ± 0.69	3.23 ± 0.93	5.36 ± 0.97
4-Heptanal, (Z)-	ND	0.09 ± 0.01	0.98 ± 0.09	6.22 ± 1.23	0.28 ± 0.02	0.18 ± 0.04	2.32 ± 0.26	1.69 ± 0.66	0.56 ± 0.08
Ketones									
2,3-Octanedione	ND	ND	0.85 ± 0.15	1.7 ± 0.56	ND	0.08 ± 0.01	1.23 ± 1.23	2.56 ± 1.56	ND
2,3-Pentanedione	0.04 ± 0.01	0.06 ± 0.02	0.03 ± 0.01	1.56 ± 0.05	1.78 ± 0.58	2.53 ± 0.04	1.03 ± 0.05	4.22 ± 1.86	3.29 ± 0.12
2-Nonanone	ND	ND	0.06 ± 0.02	0.12 ± 0.04	ND	ND	0.08 ± 0.05	0.25 ± 0.08	ND
2-Undecanone	ND	ND	0.75 ± 0.02	2.67 ± 0.66	0.96 ± 0.06	ND	1.19 ± 0.28	4.55 ± 1.32	1.89 ± 0.64
2-Heptanone	ND	ND	0.12 ± 0.02	0.89 ± 0.13	0.06 ± 0.01	ND	1.35 ± 0.61	2.46 ± 0.05	0.09 ± 0.01
Hydrocarbons									
Heptacosane	ND	ND	1.23 ± 0.13	0.85 ± 0.04	2.35 ± 0.12	2.79 ± 0.13	0.46 ± 0.02	0.77 ± 0.07	1.23 ± 0.21
Pentadecane	2.23 ± 0.14	8.63 ± 1.36	4.62 ± 0.88	3.33 ± 0.21	3.26 ± 1.02	9.59 ± 1.33	4.35 ± 0.98	2.23 ± 0.29	1.9 ± 0.06
Tetradecane	4.23 ± 0.32	2.51 ± 0.11	0.56 ± 0.02	ND	1.03 ± 0.05	1.54 ± 0.21	ND	ND	0.81 ± 0.06
Others									
Ethyl acetate	5.03 ± 0.09	7.54 ± 1.32	3.21 ± 0.35	1.22 ± 0.35	2.13 ± 0.86	3.23 ± 0.78	0.77 ± 0.12	2.27 ± 0.65	1.22 ± 0.05
Methylamine, N, N-dimethyl-	ND	ND	3.31 ± 0.09	8.02 ± 1.04	ND	3.28 ± 0.39	10.65 ± 2.65	21.23 ± 4.65	ND
Methoxy-phenyl-oxime	ND	ND	0.64 ± 0.06	0.46 ± 0.03	0.11 ± 0.02	ND	0.83 ± 0.04	2.22 ± 0.25	0.23 ± 0.03
Naphthalene	0.07 ± 0.01	0.14 ± 0.02	0.08 ± 0.01	0.07 ± 0.02	0.12 ± 0.03	0.09 ± 0.01	0.16 ± 0.04	0.06 ± 0.01	0.16 ± 0.04
Dimethyl disulfide	ND	ND	ND	0.85 ± 0.06	ND	ND	ND	1.32 ± 0.31	ND

ND means not detected.

## Data Availability

Authors can confirm that all relevant data are included in the article.
